# Role of NFκB in age-related vascular endothelial dysfunction in humans

**DOI:** 10.18632/aging.100080

**Published:** 2009-08-10

**Authors:** Anthony J. Donato, Gary L. Pierce, Lisa A. Lesniewski, Douglas R. Seals

**Affiliations:** Department of Integrative Physiology, University of Colorado, Boulder, CO 80309, USA

**Keywords:** aging, endothelial dysfunction, NFkB, oxidative stress, inflammation

Cardiovascular diseases (CVD) are the leading cause of morbidity and mortality in the United States and other industrialized societies. Older age is the major risk factor for development of CVD [1]. Emerging evidence over the past 20 years suggests that the arterial vascular endothelium plays a critical role in the development of CVD, most notably, atherosclerosis. A healthy vascular endothelium is characterized by a tightly regulated balance of pro- and anti-oxidants, vasodilators and vasoconstrictors, and pro- and anti-inflammatory molecules. A diseased or dysfunctional endothelium displays a "pro-atherogenic" phenotype, losing its tightly regulated balance and adopting a pro-oxidant/vasoconstrictor/pro-inflammatory phenotype. A hallmark of arterial endothelial dysfunction is impaired endothelial dependent dilation, which is predictive of future CVD events [1,2].

Aging leads to impaired endothelial dependent dilation associated elevated oxidative stress and a pro-inflammatory endothelial cell phenotype. Recent studies in humans by our group and by others in rodents suggest a critical role of nuclear factor κB (NFκB) in the pro-inflammatory / pro-oxidant linked suppression of endothelial dependent dilation with advancing aging [3-7]. This perspective will discuss new information concerning the role of increased NFκB signaling in mediating vascular endothelial dysfunction with aging in humans.

NFκB is an important transcription factor expressed in all mammalian cell types.  It is responsible for regulating gene expression of factors that control cell adhesion, proliferation, inflammation, redox status, and tissue specific enzymes. In arteries, NFκB is thought to promote CVD through its pro-inflammatory, pro-adhesion and pro-oxidant gene transcription. Recent evidence, however, suggests that not all NFκB-mediated gene regulation may be deleterious to the vascular system. For example, acute shear stress evoked increases in endothelial nitric oxide synthase, the enzyme that synthesizes the vascular protective molecule nitric oxide, is NFκB dependent [8]. The complexity in the control of NFκB signaling provides insight into how this transcription factor can have such diversity of regulatory responsibilities.

The NFκB activation pathway is triggered by a wide variety of stimuli including inflammatory cytokines, reactive oxygen species, lipids and mechanical forces acting on the vascular endothelial wall leading to stimulation of transmembrane receptors. This triggers intracellular signaling pathways leading to an activation of a kinase (IκK) mediated phosphorylation and degradation of the inhibitor of NFκB (IκB). This results in translocation of the NFκB heterodimer (p65/p50 subunits and, perhaps, p65, RelB, c-Rel, p50 and p52) to the nucleus where it binds to promoters of gene targets. Some potential gene targets that predispose the vasculature to endothelial dysfunction and a "proatherogenic" phenotype are pro-inflammatory molecules such as interleukin-6 (IL-6), tumor necrosis factor-α (TNF-α) monocyte chemoattractant protein 1 (MCP-1), receptor for advance glycation endproducts (RAGE) and the pro-oxidant enzyme NADPH oxidase [9-11].

Aging is associated with chronic, low-grade inflammation characterized by increases in circulating acute phase proteins C-reactive protein (CRP) and pro-inflammatory cytokines [12], such as TNF-α [13] and IL-6 [14]. Recently, we demonstrated that total NFκB protein was elevated in vascular endothelial cells collected in obese [15] and older [6] adults compared with normal weight, young controls. In a follow-up study, we determined if the age-associated increase in total NFκB expression was associated with increased signaling and downstream pro-inflammatory gene expression [5]. We found that endothelial dependent dilation was impaired in older adults and was associated with increased nuclear translocation of NFκB in their vascular endothelial cells. We also demonstrated that this increased nuclear localization was associated with a decrease in expression of IκBα. This overall activation of NFκB was associated with an increase in endothelial cell expression of the pro-inflammatory NFκB transcripts TNF-α, IL-6 and MCP-1, but not RAGE or cyclooxygenase. These results were the first to demonstrate that healthy human aging is associated with NFκB activation and selective upregulation of inflammatory proteins in the vascular endothelium. The expression of these cytokines in vascular endothelial cells was not related to plasma concentrations of TNF-α, IL-6 or CRP. This indicates that among individuals, circulating levels of these proteins cannot be used to assess the inflammatory state of the vasculature *per se*. We postulate that the development of this pro-inflammatory state in the vascular endothelium with healthy aging may play an important role in the increased susceptibility of older adults to atherosclerosis and other CVD [9].

Although these findings established that vascular inflammation developed with aging in healthy adults, our results did not provide evidence that this inflammatory state was contributing to vascular endothelial dysfunction in older adults.  We also had no insight into the mechanisms that might link inflammation to impaired endothelial function. One possibility was that NFκB activation increased oxidative stress, which, in turn, caused vascular endothelial dysfunction with aging.  Initial evidence for a role of NFκB signaling in age-associated vascular oxidative stress was provided by Donato et al. [6]. In that study, we found that total NFκB expression was positively related to nitrotyrosine, a marker of cellular oxidative stress, in vascular endothelial cells obtained from groups of young and older healthy adults.

Recently, Pierce et al. [7] provided direct evidence that NFκB activation contributes to arterial endothelial dysfunction with aging. In a group of middle-aged and older obese adults, inhibition of endothelial cell NFκB nuclear translocation was achieved by four days of high dose treatment with the non-acetylated salicylate compound, salsalate, which suppresses NFκB signaling through inhibition of the NFκB activator, IκK.  Salsalate improved endothelial dependent dilation in these older obese adults by 74% to values similar to young healthy adults. Interestingly, acute intravenous infusion of the potent antioxidant, vitamin C, improved endothelial dependent dilation during placebo but did not augment dilation further it during the Salsalate condition. Salsalate also reduced nitrotyrosine and NADPH oxidase expression in vascular endothelial cells obtained from the subjects. Taken together, these findings provide experimental support for the idea that NFκB-dependent vascular inflammation tonically impairs vascular endothelial function with aging in humans by stimulating oxidative stress.

In summary, NFκB is a key regulator of inflammation and oxidative stress. As a result of its unique ability to respond to both redox and inflammatory signaling in a cell, NFκB provides an effective "transducer" for feed forward activation of these processes. Recent findings from our laboratory provide evidence for an important role in NFκB in mediating vascular endothelial dysfunction in humans by stimulating inflammation and oxidative stress (Figure 1). Our results provide an experimental basis for future basic and clinical research studies focusing on the contribution of NFκB signaling to vascular aging. Basic research questions include the need for a greater understanding of the nuclear regulation of NFκB promoter binding and gene transcription in aging arteries. Among the key questions in this area are the mechanisms by which increases in NFκB nuclear translocation in vascular endothelial cells of older adults could lead to selective activation of genes involved in inflammation and oxidative stress. The roles of histone modification, DNA methylation, and transcription factor acetylation in such specific regulation of gene expression are worthy of attention. In cell culture, these processes modify NFκB promoter binding, but it is unknown how these mechanisms affect the vascular endothelium with aging. Clinical research directions could include determining if IκK inhibitors, such as salsalate, are viable as long term interventions to reduce tissue specific oxidative stress and inflammation with aging and other age-related disease states. Inhibiting NFκB signaling might limit the vicious cycles of inflammation and oxidative stress, in part by interrupting synergistic crosstalk between these two processes.  Thus, modulation of NFκB may be viewed as a potential therapeutic target in the prevention of arterial aging.

**Figure 1.**
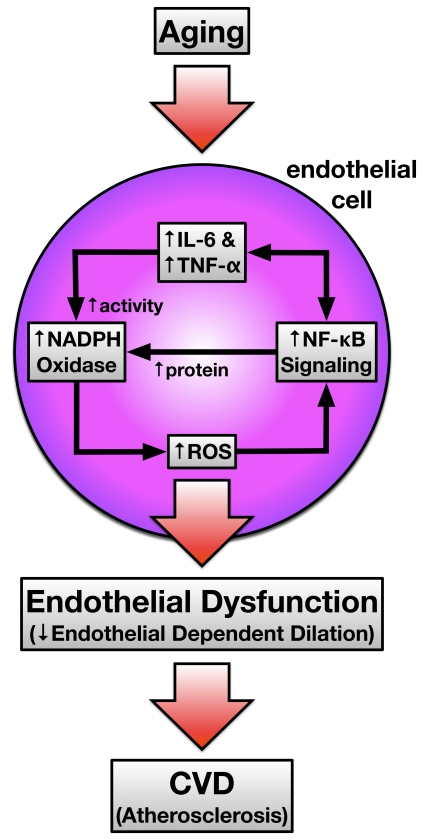
Depicts the working hypothesis of how vascular aging induces feed forward NFκB signaling that is pro-oxidant and pro-inflammatory leading to endothelial dysfunction and atherosclerosis susceptibility. IL-6, interleukin-6; TNF-α, tumor necrosis factor-α; NFκB, nuclear factor κB; ROS, reactive oxygen species; CVD, cardiovascular disease.

## References

[1] Lakatta EG, Levy D (2003). Arterial and cardiac aging: major shareholders in cardiovascular disease enterprises: Part I: aging arteries: A "set up" for vascular disease. Circulation.

[2] Widlansky ME, Gokce N, Keaney JF Jr, Vita JA (2003). The clinical implications of endothelial dysfunction. J Am Coll Cardiol.

[3] Csiszar A, Wang M, Lakatta EG, Ungvari Z (2008). Inflammation and endothelial dysfunction during aging: role of NF-kappaB. J Appl Physiol.

[4] Csiszar A, Labinskyy N, Smith K, Rivera A, Orosz Z, Ungvari Z (2007). Vasculoprotective effects of anti-tumor necrosis factor-alpha treatment in aging. Am J Pathol.

[5] Donato AJ, Black AD, Jablonski KL, Gano LB, Seals DR (2008). Aging is associated with greater nuclear NFkappaB, reduced IkappaBalpha, and increased expression of proinflammatory cytokines in vascular endothelial cells of healthy humans. Aging Cell.

[6] Donato AJ, Eskurza I, Silver AE (2007). Direct evidence of endothelial oxidative stress with aging in humans: relation to impaired endothelium-dependent dilation and upregulation of nuclear factor-kappaB. Circ Res.

[7] Pierce GL, Lesniewski LA, Lawson BR, Beske SD, Seals DR (2009). Nuclear factor-{kappa}B activation contributes to vascular endothelial dysfunction via oxidative stress in overweight/obese middle-aged and older humans. Circulation.

[8] Davis ME, Grumbach IM, Fukai T, Cutchins A, Harrison DG (2004). Shear stress regulates endothelial nitric-oxide synthase promoter activity through nuclear factor kappaB binding. J Biol Chem.

[9] de Winther MP, Kanters E, Kraal G, Hofker MH (2005). Nuclear factor kappaB signaling in atherogenesis. Arterioscler Thromb Vasc Biol.

[10] Guzik TJ, Harrison DG (2007). Endothelial NF-kappaB as a mediator of kidney damage: the missing link between systemic vascular and renal disease. Circ Res.

[11] Anrather J, Racchumi G, Iadecola C (2006). NF-kappaB regulates phagocytic NADPH oxidase by inducing the expression of gp91phox. J Biol Chem.

[12] Krabbe K, Pedersen M, Bruunsgaard H (2004). Inflammatory mediators in the elderly. Exp Gerontol.

[13] Vgontzas AN, Zoumakis M, Bixler EO (2003). Impaired nighttime sleep in healthy old versus young adults is associated with elevated plasma interleukin-6 and cortisol levels: physiologic and therapeutic implications. J Clin Endocrinol Metab.

[14] Belmin J, Bernard C, Corman B, Merval R, Esposito B, Tedgui A (1995). Increased production of tumor necrosis factor and interleukin-6 by arterial wall of aged rats. Am J Physiol.

[15] Silver AE, Beske SD, Christou DD (2007). Overweight and obese humans demonstrate increased vascular endothelial NAD(P)H oxidase-p47(phox) expression and evidence of endothelial oxidative stress. Circulation.

